# Quantification of muscle recovery in post-ICU patients admitted for acute pancreatitis: a longitudinal single-center study

**DOI:** 10.1186/s12871-024-02687-3

**Published:** 2024-09-05

**Authors:** Clarissa Hosse, Nick L. Beetz, Uli Fehrenbach, Aboelyazid Elkilany, Timo A. Auer, Bernhard Gebauer, Christian Pille, Dominik Geisel, Johannes Kolck

**Affiliations:** 1 Department of Radiology, Charité - Universitätsmedizin Berlin, Berlin, Germany; 2 Department of Anesthesiology and Intensive Care Medicine | CCM | CVK, Charité – Universitätsmedizin Berlin, Berlin, Germany; 3 Berlin Institute of Health at Charité - Universitätsmedizin Berlin, Berlin, Germany

**Keywords:** Critical care, Acute pancreatitis, Muscle wasting, Artificial intelligence, Computed tomography

## Abstract

**Objectives:**

Critically ill patients with severe pancreatitis exhibit substantial muscle wasting, which limits in-hospital and post-hospital outcomes. Survivors of critical illness undergo extensive recovery processes. Previous studies have explored pancreatic function, quality of life, and costs post-hospitalization for AP patients, but none have comprehensively quantified muscle loss and recovery post-discharge. By applying an AI-based automated segmentation tool, we aimed to quantify muscle mass recovery in ICU patients after discharge.

**Materials:**

Muscle segmentation was performed on 22 patients, with a minimum of three measurements taken during hospitalization and one clinically indicated examination after hospital discharge. Changes in psoas muscle area (PMA) between admission, discharge and follow up were calculated. T-Test was performed to identify significant differences between patients able and not able to recover their muscle mass.

**Results:**

Monitoring PMA shows muscle loss during and gain after hospitalization: The mean PMA at the first scan before or at ICU admission (TP1) was 17.08 cm², at the last scan before discharge (TP2), mean PMA was 9.61 cm². The percentage change in PMA between TP1 and TP2 ranged from − 85.42% to -2.89%, with a mean change of -40.18%. The maximum muscle decay observed during the stay was − 50.61%. After a mean follow-up period of 438.73 days most patients (81%) were able to increase their muscle mass. Compared to muscle status at TP1, only 27% of patients exhibited full recovery, with the majority still presenting a deficit of 31.96%.

**Conclusion:**

Muscle recovery in ICU patients suffering from severe AP is highly variable, with only about one third of patients recovering to their initial physical status. Opportunistic screening of post-ICU patient recovery using clinically indicated imaging and AI-based segmentation tools enables precise quantification of patients’ muscle status and can be employed to identify individuals who fail to recover and would benefit from secondary rehabilitation. Understanding the dynamics of muscle atrophy may improve prognosis and support personalized patient care.

## Introduction

Among the various gastrointestinal conditions leading to hospitalization, acute pancreatitis (AP) is one of the most prevalent. The severity of AP varies significantly, ranging from self-limiting cases to those with rapidly fatal outcomes [1, 2]. In severe cases, patients often require prolonged hospitalization and admission to the intensive care unit (ICU) for comprehensive management [3]. While a multidisciplinary approach remains essential, the treatment strategy for severe AP has evolved to prioritize aggressive intensive care over early surgical intervention [4].

A common complication among ICU patients is severe loss of muscle mass, which, combined with loss of muscle function, is referred to as ICU-acquired weakness (ICUAW) [5, 6]. Muscle wasting typically begins in the first days after admission to the ICU and progresses over time. The extent of muscle loss correlates with the severity of the underlying illness and the length of hospital stay and it is particularly high in septic patients [7]. In general, survivors of critical illness generally undergo an extended and resource-intensive recovery process. Previous studies have predominantly focused on pancreatic function, quality of life, and associated costs following hospitalization for AP. However, to our knowledge, no comprehensive study has addressed the quantification of muscle loss and the subsequent rebuilding of muscle mass post-discharge [8, 9].

The aim of this study was to quantify the recovery of muscle mass in intensive care patients admitted for acute pancreatitis following hospitalization.

## Materials and methods

### Study design and patient population

In this retrospective cohort study, we examined body composition metrics in critically ill patients admitted for severe pancreatitis. The study received approval from the Institutional Review Board (Internal registration number: EA4/152/20) and adhered to the principles outlined in the Declaration of Helsinki. The ethics committee waived the need to obtain patient consent. We conducted a retrospective search in our database for adult patients (aged > 18 years) who were admitted to the ICU of our university hospital between 2012 and 2022 with pancreatitis. Inclusion criteria required a clinically or morphologically confirmed diagnosis of pancreatitis, a minimum ICU stay of 10 days, and the availability of three serial computed tomography (CT) datasets of the abdomen during hospitalization. To ensure a comparable baseline assessment, only patients who underwent a CT scan prior to admission or within 6 days of ICU admission were included. The ethics committee waived the requirement for patient consent.

### Segmentation of tissue compartments

The quantification of patient tissue compartments was conducted using an AI-based automated image segmentation tool integrated into the hospital’s Picture Archiving and Communication System (PACS) software (Visage version 7.1., Visage Imaging GmbH, Berlin, Germany), a method validated in prior studies [10–12]. Following the automated identification of the third lumbar vertebra (L3) level, the system performed segmentation to categorize tissues into subcutaneous fat (SAT), skeletal muscle area (SMA), visceral fat (VAT), and psoas muscle area (PMA). Subsequently, the software computed the areas in square centimeters (cm^2^) for each of these components. An experienced radiologist reviewed each automated segmentation and applied manual corrections if necessary.

### Definition of obesity, Sarcopenia and muscular recovery

Obesity was defined as internationally recognised by a BMI threshold of > 30. Sarcopenia was determined using gender-specific cut-offs; SMA < 34.3 cm² for women, and < 45.4 cm² for men, based on established literature [13]. In our study, we defined muscular recovery as the restoration of muscle area (PMA) to the level observed at hospital admission.

### Statistics

Statistical analysis utilized SPSS software version 25.0 (IBM; New York, USA). Descriptive statistics are presented as means and standard deviations. Changes in muscle mass between admission, discharge and follow up were calculated by dividing the occurred PMA change at discharge or follow-up by the respective baseline PMA at admission or discharge. The Mann-Whitney U Test (for non-normally distributed data) and the Chi-Squared Test were used to compare patient subgroups. A sample size was not calculated. All *p*-values less than 0.05 were considered statistically significant.

## Results

### Demographic data and hospitalization

Out of 330 patients admitted to the designated ICUs during the study period, 230 were excluded due to not meeting inclusion criteria, lacking clinical data, or having fewer than three relevant CT examinations for muscle segmentation. One hundred patients meeting the criteria were included, among whom 41 died. The data of the long-term monitoring have previously been published [14]. Among the survivors, 22 patients received CT imaging after discharge and were enrolled in the study. The cohort consisted of 22 patients, with a mean age of 56.14 ± 11.5 years. The average BMI for the cohort was 25.76 ± 5.14 kg/m², with individual values ranging. Notably, the majority of patients were overweight upon admission (77.27%), and 45.45% were classified as obese. Sarcopenia was present in 40.90% of individuals. Average time from hospital to ICU admission was 6.68 ± 6.51 days. Hospital stays averaged 177 ± 86.11 days while ICU stays averaged 109.68 ± 58.2 days). Initial Sepsis-related organ failure assessment score (SOFA) was 6.16 ± 2.34. The main findings are summarized in Table 1.

### Muscle loss during and gain after hospitalization

**Fig. 1:**
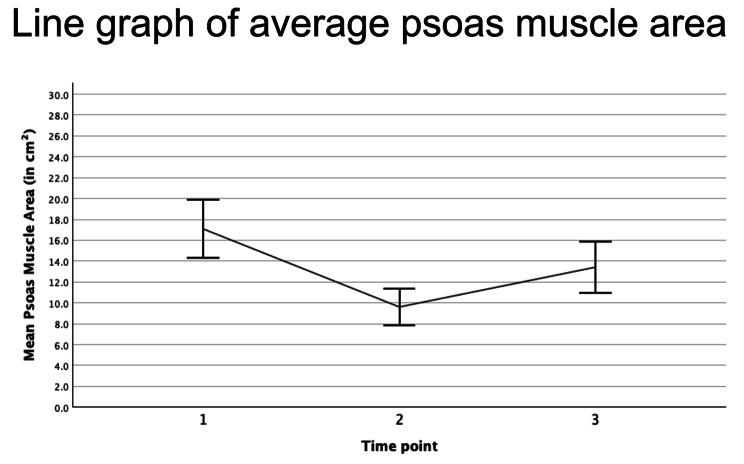
Line graph of average psoas muscle area of all 22 patients at ICU admission (1: 17.08 cm²), after a statistically significant decay (*p* < 0.001) prior to discharge (2: 9.61 cm².) and at follow up (3: 13.42 cm², *p* = 0.012)

The mean PMA at the first scan prior to or at ICU admission (TP1), was 17.08 ± 6.15 cm², spanning from 7.68 to 30.04 cm². At the last scan prior to discharge (TP2), the mean PMA was 9.61 ± 3.81 cm², ranging from 2.87 to 22.41 cm². The percentage change in PMA between TP1 and TP2 among the patients was − 40.18 ± 22.78%. While the maximum muscle decay patients experienced during their stay, assessed over all available scans, was higher at -50.61 ± 13.02%. Follow-up periods post-discharge averaged at 478.14 ± 333 days. AT follow up (TP3), the mean PMA was 13.42 ± 5.5 cm², with a range from a minimum of 4.94 to 24.38 cm² (Fig. 1).

### Subgroup analysis

Statistical analysis unveiled noteworthy distinctions between patients demonstrating muscle recovery and those who did not. Notably, the recovering patients manifested a higher prevalence of obesity and consistently higher PMA across all three time points. Moreover, among individuals who attained full recovery to their initial physical state, a lesser proportion were female, as delineated in Table 2.

**Table 1:** Overview of enrolled patients. TP1 = Time point 1: first CT within prior or within 6 days of ICU admission. TP2 = time point 2: last CT prior to discharge. TP3 = Time point 3: follow up CT

ID	Age	Sex	BMI	Hospital stay in days	ICU stay in days	PMA TP1	PMA TP2	PMA TP3	Δ PMA TP 1/2	Peak PMA loss during hospital stay	Time to follow up	Δ PMA TP2/3
1	72	M	25.56	336	192	16.71	5.05	10.17	-69.78	-69.98%	99	101.39%
2	55	M	37.81	51	38	20.52	12.46	22.41	-39.28%	-39.28%	1075	79.86%
3	63	M	16.67	139	111	11.28	10.55	12.42	-6.47	-39.80%	685	17.73%
4	58	F	23.98	133	100	7.68	6.49	7.45	-15.49%	-15.49%	587	14.79%
5	39	M	29.41	88	75	18.55	12.95	11.96	-30.19%	-30.19%	808	-7.64%
6	57	F	23.67	288	126	10.88	4.42	7.72	-59.38%	-59.38%	554	74.66%
7	39	M	27.13	76	58	18.97	7.06	11.84	-62.78%	-62.78%	803	67.71%
8	79	F	25.39	235	119	16.51	13.68	11.26	-17.14	-44.46%	104	-17.69%
9	59	M	23.84	98	83	22.40	14.73	14.81	-34.24	-36.47%	27	0.54%
10	53	M	28.40	308	208	26.65	11.25	11.65	-57.79	-71.33%	49	3.56%
11	50	M	19.37	142	56	16.7	5.74	17.8	-65.63%	-65.63%	984	210.10%
12	59	M	24.97	300	260	23.06	16.80	23.5	-27.15	-31.31%	450	39.88%
13	57	M	21.91	158	149	13.06	10.68	18.26	-18.22	-50.92%	145	70.97%
14	35	M	27.10	192	105	14.9	12.62	12.95	-15.30	-60.67%	242	2.61%
15	67	M	23.57	60	10	13.43	9.28	8.13	-30.90	-35.44%	104	-12.39%
16	46	F	20.98	102	49	16.59	10.11	16.31	-39.06	-45.27%	445	61.33%
17	61	M	24.22	137	130	13.84	5.73	6.33	-58.60	-71.39%	124	10.47%
18	63	F	37.38	320	135	9.09	2.87	4.94	-68.43%	-68.43%	615	72.13%
19	52	M	25.93	170	77	29.61	13.84	20.29	-53.26	-55.45%	114	46.60%
20	78	F	24.39	174	148	9.01	8.75	10.95	-2.89	-27.08%	634	25.14%
21	49	F	20.76	152	119	16.24	11.93	9.62	-26.54	-47.17%	107	-19.36%
22	44	M	34.19	226	50	30.04	4.38	24.38	-85.42%	-85.42%	897	456.62%
Ø	56.14		25.76	177	109	17.08	9.61	13.42	-40.18	-50.61%	439	59.05%

**Table 2:** Results of t-test comparing individuals who showed recovery in muscle area (= 1) with those who did not (= 0), and comparison of those who recovered to their initial status at ICU admission (= 1) with those who did not (= 0)

Recovery after discharge		*N*	Mean	SD	*p*		Recovery to TP1		*N*	Mean	SD	*p*	
Female Gender	1	18	28%	46%	0.616		Female Gender	1	6	17%	41%	0.565	
	0	4	50%	58%				0	16	38%	50%		
Age	1	18	55.61	10.62	0.831		Age	1	6	60.33	9.66	0.267	
	0	4	58.50	17.91				0	16	54.56	12.38		
Obesity	1	18	17%	51%	1.00		Obesity	1	6	13%	52%	1.00	
	0	4	0%	0%				0	16	17%	50%		
Sarcopenia	1	18	44%	51%	0.655		Sarcopenia	1	6	50%	55%	0.616	
	0	4	25%	50%				0	16	38%	50%		
PMA at TP1	1	18	17.27	6.93	0.837		PMA at TP1	1	6	15.61	5.47	0.641	
	0	4	16.18	2.10				0	16	17.63	6.67		
PMA at TP2	1	18	9.09	4.06	0.195		PMA at TP2	1	6	10.83	3.70	0.541	
	0	4	11.96	1.93				0	16	9.15	3.98		
PMA at TP3	1	18	14.12	5.98	0.262		PMA at TP3	1	6	17.56	5.09	**0.040**	
	0	4	10.24	1.72				0	16	11.86	5.13		
Time btw. TP2 and TP3	1	18	473.83	339.05	0.342		Time btw. TP2 and TP3	1	6	662.17	342.87	**0.040**	
	0	4	280.75	351.50				0	16	354.94	310.62		
Hospital.in days	1	18	186.11	89.46	0.342		Hospital.in days	1	6	160.67	80.55	0.802	
	0	4	133.75	77.71				0	16	182.56	92.59		

## Discussion

The primary aim of this study was to rigorously quantify alterations in muscle mass among ICU patients diagnosed with acute pancreatitis post-discharge. To our knowledge, we present the first quantitative follow-up data on muscle decay by utilizing an AI-based tool for PMA segmentation in clinically indicated CT scans. Our data provides several important insights. Firstly, opportunistic screening for muscle area proves to be a sufficient tool for monitoring the recovery of ICU patients by quantifying their gains and losses. Secondly, the average muscle loss during hospitalization in the patient population suffering from severe pancreatitis was high, at 40.18% ± 23.31% and showed high patient individual variability. Thirdly, after a mean follow-up period of 438.73 days most patients (81%) were able to increase their muscle mass, with mean gains of 75.33% ± 107.47% compared to the last obtained status during hospitalization. Notably, two patients already exhibited muscle gain as signs of recovery during hospitalization, being discharged with an overall loss of less than 10% (ID 3 and ID 21). However, when comparing muscle status to ICU admission, only 27% of patients exhibited recovery, with the majority still presenting a deficit of 31.96% ± 15.92% PMA at follow-up. Additionally, among the very few individuals presenting with a full recovery (ID 2, 3, 11, 12, 13, and 20), it is noticeable that some had more muscle area at the follow-up time than at the initial imaging. This difference is primarily attributed to muscle loss that occurred before the initial imaging study. Finally, in some patients, muscle deterioration continues even after more than 100 days post-discharge (ID 15 and ID 21).

The long-term health outcomes of individuals who survive ICU stays have become a growing concern in recent years. This is especially true as the number of ICU survivors rises due to increased demand for critical care and reduced ICU mortality rates. Survivors of intensive care often encounter post-intensive care sequelae, which are commonly categorized under the term “post-intensive care syndrome” (PICS). This syndrome encompasses cognitive, psychological, and/or physical impairments [15, 16]. One component of PICS is intensive care acquired weakness (ICUAW), which is marked by severe loss of muscle mass and function [5, 17, 18]. The consequences of PICS and ICUAW on quality of life, health-related costs and hospital readmissions are real public health problems.

Although the pathophysiology of muscle atrophy and dysfunction during critical illness seems to be partially elucidated, the mechanisms involved in persistent muscle dysfunction resulting in impaired physical recovery after ICU discharge remain poorly understood [19].

Typically, women and older individuals exhibit a higher susceptibility to experiencing inadequate functional recuperation following critical illness. The variances between genders still lack clarity but could potentially be linked to the lesser pre-existing muscle mass. Generally, low muscle mass has been linked to less favorable pre-ICU health condition and post-ICU outcomes [20, 21]. In our study the recovery to the initial muscle status was significantly associated with the time to follow-up. While gender, initial muscularity, obesity and sarcopenia were not associated with the recovery, in this relatively small cohorts.

Assessing muscle mass in the ICU is crucial, as a recent study underscored, where researchers applied bio-impedance analysis (BIA) to evaluate muscle decay and found a significant correlation between inadequate nutritional intake and increased muscle wasting. This highlights the need for early and adequate nutritional support to mitigate muscle loss and enhance recovery [22].

### Limitations

Given the retrospective nature of our study, a selection bias is inevitable. The retrospective design presents challenges in establishing causality between the degree and timing of muscle recovery, as does the absence of data on post-discharge rehabilitation programs. In addition, the comparable small study population restricts the generalizability of our results. Future prospective studies with a more robust design could provide a clearer understanding of the dynamics of muscle loss and recovery rates.

## Conclusion

Individuals who have experienced acute pancreatitis requiring intensive care often endure significant muscle depletion. The recuperation from these ICU stays varies widely among patients, with a majority displaying persistent deficits in muscle mass. Opportunistic screening of post-ICU patient recovery through the segmentation of clinically indicated transactional imaging enables precise quantification of patients’ muscle status and may be employed to identify individuals who could benefit from secondary rehabilitation.

## Data Availability

All data generated or analysed during this study are included in this published article [and its supplementary information files].

## Abbreviations

AI: Artificial intelligence
BMI: Body mass index
CT: Computed tomography
ICU: Intensive care unit
ICUAW: ICU-acquired weakness
PACS: Picture archiving and communication system
PICS: Post-intensive care syndrome
PMA: Psoas muscle area
SAT: Subcutaneous adipose tissue
SMA: Skeletal muscle area
VAT: Visceral adipose tissue
